# Risk prediction models of gestational diabetes mellitus before 16 gestational weeks

**DOI:** 10.1186/s12884-022-05219-4

**Published:** 2022-12-01

**Authors:** Yiling Wei, Andong He, Chaoping Tang, Haixia Liu, Ling Li, Xiaofeng Yang, Xiufang Wang, Fei Shen, Jia Liu, Jing Li, Ruiman Li

**Affiliations:** 1 Department of Obstetrics and Gynecology, The First Affiliated Hospital of Jinan University, Guangzhou, 510630 China; 2 Department of Obstetrics and Gynecology, The Third Affiliated Hospital of Guangzhou Medical University, Guangzhou, 510150 China; 3 Department of Obstetrics and Gynecology, Jiangmen Maternity and Child Health Care Hospital, Jiangmen, 529000 China

**Keywords:** Gestational diabetes mellitus, Prediction model, Nomogram, Early pregnancy

## Abstract

**Background:**

Gestational diabetes mellitus (GDM) can lead to adverse maternal and fetal outcomes, and early prevention is particularly important for their health, but there is no widely accepted approach to predict it in the early pregnancy. The aim of the present study is to build and evaluate predictive models for GDM using routine indexes, including maternal clinical characteristics and laboratory biomarkers, before 16 gestational weeks.

**Methods:**

A total of 2895 pregnant women were recruited and maternal clinical characteristics and laboratory biomarkers before 16 weeks of gestation were collected from two hospitals. All participants were randomly stratified into the training cohort and the internal validation cohort by the ratio of 7:3. Using multivariable logistic regression analysis, two nomogram models, including a basic model and an extended model, were built. The discrimination, calibration, and clinical validity were used to evaluate the models in the internal validation cohort.

**Results:**

The area under the receiver operating characteristic curve of the basic and the extended model was 0.736 and 0.756 in the training cohort, and was 0.736 and 0.763 in the validation cohort, respectively. The calibration curve analysis showed that the predicted values of the two models were not significantly different from the actual observations (*p* = 0.289 and 0.636 in the training cohort, *p* = 0.684 and 0.635 in the internal validation cohort, respectively). The decision-curve analysis showed a good clinical application value of the models.

**Conclusions:**

The present study built simple and effective models, indicating that routine clinical and laboratory parameters can be used to predict the risk of GDM in the early pregnancy, and providing a novel reference for studying the prediction of GDM.

**Supplementary Information:**

The online version contains supplementary material available at 10.1186/s12884-022-05219-4.

## Background

Gestational diabetes mellitus (GDM) is one of the most common complications of pregnancy, affecting about 2 to 25% of pregnancies worldwide [1]. GDM is defined as glucose intolerance with onset or first recognized during pregnancy [2]. GDM is associated with multiple adverse outcomes for both mother and baby during and beyond pregnancy. For pregnant women, GDM is associated with increased risk of cesarean section, gestational hypertension, and polyhydramnios, and even about 50% of them develop type II diabetes mellitus (DM) within 5 years [3, 4]. As for fetus, the risk of stillbirth, preterm labor, and macrosomia may be increased; moreover, the exposure of embryos or fetuses to a hyperglycemic environment in the uterus may result in increased risk of obesity, diabetes, metabolic syndrome, and other cardiometabolic disorders in the further [5, 6].

Currently, there is no clinical guideline and consensus about testing GDM before 75 g oral glucose tolerance test (OGTT) between 24 and 28 weeks of pregnancy [7]. However, epigenetic changes and abnormal fetal growth before the diagnosis of GDM create a limited time frame for physician intervention [8]. On one hand, testing performed universally on pregnant women may increase a burden to the women without GDM. On the other hand, selective testing in women at high risk for diabetes, GDM may be underdiagnosed [9, 10]. However, there is no international consensus for a preferred screening method or detection strategy. The current guidelines only suggest selective testing based on the maternal clinical risk factors, but this binary approach is limited by its poor sensitivity and specificity. For GDM prediction, some novel biomarkers have been reported as potential predictors, including plasma fatty acid-binding protein 4, sex hormone binding globulin, microRNA, and exosome [11–14], but their low availabilities in clinical practice limit their application. Studies have showed that routine biochemical measures, such as ferritin, glycosylated hemoglobin, triglycerides, and pregnancy-associated plasma protein A (PAPP-A), may be the predictive factors for GDM in the early pregnancy [15–18]. However, these novel biochemical measures have not been thoroughly examined and the equations are complex, which make them difficult to be used in clinic. Several studies have utilized electronic health records or laboratory tests to construct prediction models for GDM [19, 20], which can be used to predict this disease using the medical data of the early pregnancy. However, there is no widely accepted early prediction model for GDM until now.

In the present study, we selected some potential GDM-related parameters from clinical routine examinations to construct a model for predicting GDM based on Nomogram, which may be a valuable supplement and be helpful for improving the screening strategy for GDM.

## Methods

### Study design

The present study was performed based on the retrospective data, involving 2895 pregnant women who delivered a singleton at the First Affiliated Hospital of Jinan University and Jiangmen Maternity and Child Health Care Hospital from January 2019 to December 2021, which was approved by the Scientific and Ethics Review Committees of the two hospitals. Notably, the Scientific and Ethics Review Committees had waived informed consent for the study as the nature of the present study is retrospective. Two models were built due to the differences of laboratory biomarkers among the two hospitals: the basic model (*n* = 2895) included all the laboratory biomarkers, including white blood cell count (WBC), neutrophil percentage (NEUT), red blood cell count (RBC), hemoglobin (HGB), hematocrit (HCT), platelet count (PLT), mean platelet volume (MPV), alanine aminotransferase (ALT), aspartate aminotransferase (AST), fasting blood glucose (FBG), creatinine, uric acid, prothrombin time (PT), activated partial thromboplastin time (APTT), fibrinogen (FIB), and PAPP-A, in the two hospitals, while the extended model (*n* = 2116) added several characteristics, including PT, APTT, FIB, and PAPP-A, which were potentially associated with GDM according to our previous preliminary analysis and collected only from the First Affiliated Hospital of Jinan University. All experiments were carried out in accordance with relevant guidelines and regulations.

### Study participants

The inclusion criteria included: (1) age ≥ 18 years, (2) singleton pregnancy, and the exclusion criteria were as follow: (1) primary disease, such as type I or type II DM, chronic hypertension, congenital heart disease, thyroid disorders, and liver or renal insufficiency, (2) incomplete case data records. The OGTT were performed on all participants for the diagnosis of GDM. A pregnant woman was considered to be normotensive if she had a negative OGTT result and had no other pregnancy complications, such as pregnancy hypertension, oligohydramnios, or placental abruption. Additionally, GDM should be diagnosed if one or more values met the International Association of Diabetes and Pregnancy Study Groups’ criteria in 2010 [21]: 75 g OGTT FBG ≥ 5.1 mmol/L, 1 h plasma glucose ≥ 10 mmol/L, or 2 h plasma glucose ≥ 8.5 mmol/L.

### Maternal characteristics and laboratory biomarkers

Maternal characteristics and laboratory parameters were obtained from electronic medical records at the first obstetric visit before 16 gestational weeks. Maternal characteristics included age, pre-pregnancy body mass index (BMI), parity, previous GDM history, family history of DM, polycystic ovary syndrome (PCOS), history of adverse pregnancy (such as spontaneous abortions ≥ 3 times, unexplained fetal death at > 20 weeks of gestation, and fetal anomalies despite normal karyotype), and macrosomia in previous gestation. Included laboratory parameters have been mentioned above.

### Statistical analysis

Statistical analysis was performed using R 4.0.1 software. Nonnormal distribution continuous data were presented as median and interquartile ranges, and normal distribution continuous data were shown as mean ± standard deviation. Continuous variables were compared with the Welch’s t-test or the Wilcoxon rank sum test. Categorical variables were summarized by counts and percentages and compared by either the Pearson’s chi-square test or Fisher’s exact test. Next, multivariable logistic regression analysis was used to assess the significance of each variable to explore risk factors of GDM. A collinearity test based on a variance inflation factor was performed on the logistic regression models used in the training sets. All participants were randomly stratified into the training cohort and the internal validation cohort by the ratio of 7:3. Internal validation was performed using a bootstrapping with 1000 random samples drawn with replacement. Comparison of the present prediction models and a previously published model was carried out. Area under the receiver operating characteristic (ROC) curve (AUC) was applied to evaluate the differentiation ability of the models. The Hosmer–Lemeshow test was used to reflect the calibration of each model. Moreover, the net reclassification improvement (NRI) and integrated discrimination improvement (IDI) were used to compare the accuracy and comprehensiveness of the models. The consistency of GDM probabilities predicted using the nomogram with the actual situation was evaluated by drawing calibration plots. Finally, we used the decision-curve analysis (DCA) to evaluate the clinical validity of the models [20, 21]. Statistical significance was defined as *p* ≤ 0.05.

## Results

### Characteristics of study population

The basic and extended populations respectively included 2895 (1601 normal pregnant women and 1294 patients with GDM) and 2116 pregnant women (1206 normal pregnant women and 910 patients with GDM). Additionally, there were 2026 and 869 participants in the training and validation cohorts in the basic model, and 1481 and 635 participants in the training and validation cohorts in the extended model, respectively. Maternal characteristics and laboratory biomarkers of the basic model and the extended model are shown in Tables 1 and 2, respectively. There was no significant difference on these maternal characteristics, including age, pre-pregnancy BMI, parity, previous GDM history, family history of DM, PCOS, history of adverse pregnancy, and history of macrosomia, and laboratory biomarkers, including WBC, NEUT, RBC, HGB, HCT, PLT, MPV, ALT, AST, FBG, creatinine, uric acid, PT, APTT, FIB, and PAPP-A, between the training and the validation cohorts in both the basic and the extended populations (*p* > 0.05).

**Table 1:** Clinical characteristics and laboratory biomarkers among the basic population

Variable	Training cohort (n = 2026)	Validation cohort (n = 869)	*p*
Age (year)	30.0 (27.0–32.0)	30.0 (27.0–32.0)	0.753
Nulliparous, n (%)			0.443
No	1254 (61.9)	524 (39.7)	
Yes	772 (38.1)	345 (60.3)	
History of GDM, n (%)			0.276
No	1934 (95.5)	838 (96.4)	
Yes	92 (4.5)	31 (3.6)	
History of macrosomia, n (%)			0.099
No	1992 (98.3)	862 (99.2)	
Yes	34 (1.7)	7 (0.8)	
History of abnormal pregnancy, n (%)			0.474
No	1943 (95.9)	828 (95.3)	
Yes	83 (4.1)	41 (4.7)	
Family history of DM, n (%)			0.709
No	2000 (98.7)	860 (99.0)	
Yes	26 (1.3)	9 (1.0)	
PCOS, n (%)			0.911
No	2000 (98.7)	859 (98.8)	
Yes	26 (1.3)	10 (1.2)	
Pre–pregnancy BMI (kg/m^2^)	20.6 (18.9–22.7)	20.5 (18.9–22.6)	0.875
WBC (10^9^/L)	8.9 (7.6–10.4)	8.8 (7.7–10.1)	0.420
NEUT (%)	72.3 (68.1–75.9)	72.1 (68.1–76.0)	0.881
RBC (10^12^/L)	4.0 (3.8–4.3)	4.1 (3.8–4.3)	0.316
HGB (g/L)	120.5 (114.0–127.0)	120.4 (114.0–128.0)	0.872
HCT (%)	36.0 (34.0–37.9)	36.0 (34.0–38.0)	0.457
PLT (10^9^/L)	241.5 (207.7–278.7)	238.5 (205.6–273.0)	0.277
MPV (fL)	9.6 (8.6–10.4)	9.5 (8.6–10.4)	0.977
ALT (U/L)	12.1 (9.2–18.0)	12.0 (9.5–17.0)	0.591
AST (U/L)	16.0 (13.5–19.0)	16.0 (13.6–18.4)	0.494
Creatinine (μmol/L)	45.7 (41.0–51.0)	46.0 (41.2–51.4)	0.283
Uric acid (μmol/L)	239.0 (202.5–278.2)	233.0 (203.0–270.0)	0.202
FBG (mmol/L)	4.5 (4.2–4.9)	4.5 (4.3–4.9)	0.405

*Abbreviations*: *GDM* Gestational diabetes mellitus, *DM* Diabetes mellitus, *PCOS* Polycystic ovary syndrome, *BMI* Body mass index, *WBC* White blood cell count, *NEUT* Neutrophil percentage, *RBC* Red blood cell count, *HGB* Hemoglobin, *HCT* Hematocrit, *PLT* Platelet count, *MPV* Mean platelet volume, *ALT* Alanine aminotransferase, *AST* Aspartate aminotransferase, *FBG* Fasting blood glucose

**Table 2:** Clinical characteristics and laboratory biomarkers among the extended population

Variable	Training cohort (n = 1481)	Validation cohort (n = 635)	*p*
Age (year)	30.0 (27.0–32.0)	29.0 (27.0–32.0)	0.438
Nulliparous, n (%)			0.147
No	898 (60.6)	407 (64.1)	
Yes	583 (39.4)	228 (35.9)	
History of GDM, n (%)			0.109
No	1406 (94.9)	591 (93.1)	
Yes	75 (5.1)	44 (6.9)	
History of macrosomia, n (%)			0.999
No	1456 (98.3)	625 (98.4)	
Yes	25 (1.7)	10 (1.6)	
History of abnormal pregnancy, n (%)			0.845
No	1427 (96.4)	610 (96.1)	
Yes	54 (3.6)	25 (3.9)	
Family history of DM, n (%)			0.911
No	1458 (98.4)	624 (98.3)	
Yes	23 (1.6)	11 (1.7)	
PCOS, n (%)			1.000
No	1457 (98.4)	624 (98.3)	
Yes	24 (1.6)	11 (1.7)	
Pre-pregnancy BMI (kg/m^2^)	20.5 (18.9–22.7)	20.6 (18.9–22.6)	0.631
WBC (10^9^/L)	9.0 (7.8–10.4)	9.2 (7.8–10.5)	0.248
NEUT (%)	72.8 (68.7–76.5)	72.9 (68.9–76.6)	0.384
RBC (10^12^/L)	4.0 (3.8–4.3)	4.0 (3.8–4.3)	0.816
HGB (g/L)	121.1 (114.3–128.0)	121.0 (114.0–128.0)	0.950
HCT (%)	36.1 (34.0–38.0)	36.0 (33.8–37.9)	0.667
PLT (10^9^/L)	235.0 (203.0–270.5)	233.0 (202.5–271.0)	0.826
MPV (fL)	9.1 (8.3–10.1)	9.2 (8.3–10.0)	0.494
ALT (U/L)	12.0 (9.0–19.0)	12.0 (9.0–17.0)	0.186
AST (U/L)	16.0 (13.9–20.0)	16.0 (13.5–19.0)	0.190
Creatinine (μmol/L)	46.6 (41.4–53.0)	46.0 (41.5– 52.5)	0.416
Uric acid (μmol/L)	237.0 (204.0–275.0)	235.0 (201.0–275.3)	0.793
FBG (mmol/L)	4.5 (4.2–4.9)	4.5 (4.2–4.9)	0.344
PT (s)	12.3 (10.9–12.8)	12.3 (10.1–12.8)	0.812
APTT (s)	32.6 (29.0–34.9)	32.3 (28.8–34.6)	0.213
FIB (mg/dL)	4.1 (3.7–4.5)	4.1 (3.7–4.5)	0.775
PAPP-A (MoM)	1.1 (0.7–1.6)	1.1 (0.7–1.6)	0.330

*Abbreviations*: *GDM* Gestational diabetes mellitus, *DM* Diabetes mellitus, *PCOS* Polycystic ovary syndrome, *BMI* Body mass index, *WBC* White blood cell count, *NEUT* Neutrophil percentage, *RBC* Red blood cell count, *HGB* Hemoglobin, *HCT* Hematocrit, *PLT* Platelet count, *MPV* Mean platelet volume, *ALT* Alanine aminotransferase, *AST* Aspartate aminotransferase, *FBG* Fasting blood glucose, *PT* Prothrombin time, *APTT* Activated partial thromboplastin time, *FIB* Fibrinogen, *PAPP-A* Pregnancy associated plasma protein A

### Prediction risk factors of identification

Potential clinical characteristics and laboratory biomarkers for GDM were explored by the univariable logistic regression (Tables 3 and 4). The age of the participants in the GDM group was significantly older than the normal group (*p* < 0.001). Compared with the normal group, the proportion of nulliparous of the GDM group was significantly lower (*p* < 0.001), and the proportions of history of macrosomia, history of GDM, family history of DM, and PCOS of the GDM group were significantly higher (*p* < 0.05). The pre-pregnancy BMI of the GDM group was higher than that of the normal group (*p* < 0.001). The levels of WBC, RBC, HCT, PLT, MPV, ALT, uric acid, and FBG in the GDM group were significantly higher than those in the normal group, but the level of creatinine of the GDM group was significantly lower (*p* < 0.05). In the extended population, the levels of PT, APTT, and PAPP-A of the GDM group were significantly lower than those of the normal group, but the level of FIB of the GDM group was significantly higher (*p* < 0.001). HGB level of the GDM group was higher than that of the normal group in the extended population (*p* = 0.005), while there was no significant difference in the basic population (*p* = 0.209).

**Table 3:** Univariable analysis of the GDM and the normal groups among the basic population

Variable	GDM group (n = 909)	Normal group (n = 1117)	*p*
Age (year)	31.0 (28.0–33.0)	29.0 (27.0–31.0)	< 0.001
Nulliparous, n (%)			< 0.001
No	610 (67.1)	644 (42.3)	
Yes	299 (32.9)	473 (57.7)	
History of GDM, n (%)			< 0.001
No	829 (91.2)	1105 (98.9)	
Yes	80 (8.8)	12 (1.1)	
History of macrosomia, n (%)			0.001
No	884 (97.2)	1108 (99.2)	
Yes	25 (2.8)	9 (0.8)	
History of abnormal pregnancy, n (%)			0.063
No	864 (95.0)	1080 (96.7)	
Yes	45 (5.0)	37 (3.3)	
Family history of DM, n (%)			< 0.001
No	887 (97.6)	1113 (99.6)	
Yes	22 (2.4)	4 (0.4)	
PCOS, n (%)			0.006
No	890 (97.9)	1110 (99.4)	
Yes	19 (2.1)	7 (0.6)	
Pre–pregnancy BMI (kg/m^2^)	21.3 (19.4–23.5)	20.1 (18.5–21.9)	< 0.001
WBC (10^9^/L)	9.2 (7.9– 10.8)	8.7 (7.4–10.0)	< 0.001
NEUT (%)	72.5 (68.2–76.0)	72.2 (67.9–75.9)	0.419
RBC (10^12^/L)	4.1 (3.8–4.3)	4.0 (3.8–4.3)	0.014
HGB (g/L)	121.0 (114.0–127.0)	120.3 (113.0–127.0)	0.209
HCT (%)	36.0 (34.0–37.9)	35.8 (33.7–37.9)	0.012
PLT (10^9^/L)	245.4 (210.0–286.6)	237.6 (204.0–272.0)	< 0.001
MPV (fL)	9.7 (8.8–10.4)	9.4 (8.4–10.4)	< 0.001
ALT (U/L)	12.9 (9.6–18.9)	12.0 (9.0–17.7)	0.025
AST (U/L)	15.8 (13.3–19.0)	16.0 (13.8–19.9)	0.494
Creatinine (μmol/L)	45.0 (40.6–49.4)	46.0 (41.2–52.2)	< 0.001
Uric acid (μmol/L)	245.0 (209.0–287.0)	233.0 (198.0–270.0)	< 0.001
FBG (mmol/L)	4.6 (4.3–5.0)	4.5 (4.2–4.8)	< 0.001

*Abbreviations*: *GDM* Gestational diabetes mellitus, *DM* Diabetes mellitus, *PCOS* Polycystic ovary syndrome, *BMI* Body mass index, *WBC* White blood cell count, *NEUT* Neutrophil percentage, *RBC* Red blood cell count, *HGB* Hemoglobin, *HCT* Hematocrit, *PLT* Platelet count, *MPV* Mean platelet volume, *ALT* Alanine aminotransferase, *AST* Aspartate aminotransferase, *FBG* Fasting blood glucose

**Table 4:** Univariable analysis of the GDM and the normal groups among the extended population

Variable	GDM group (n = 637)	Normal group (n = 844)	*p*
Age (year)	30.0 (28.0–33.0)	29.0 (27.0–31.0)	< 0.001
Nulliparous, n (%)			< 0.001
No	432 (67.8)	466 (55.2)	
Yes	205 (32.2)	378 (44.8)	
History of GDM, n (%)			< 0.001
No	573 (90.0)	833 (98.7)	
Yes	64 (10.0)	11 (1.3)	
History of macrosomia, n (%)			0.005
No	619 (97.2)	837 (99.2)	
Yes	18 (2.8)	7 (0.8)	
History of abnormal pregnancy, n (%)			0.182
No	609 (95.6)	818 (96.9)	
Yes	28 (4.4)	26 (3.1)	
Family history of DM, n (%)			< 0.001
No	615 (96.5)	841 (99.6)	
Yes	22 (3.5)	3 (0.4)	
PCOS, n (%)			0.003
No	619 (97.2)	838 (99.3)	
Yes	18 (2.8)	6 (0.7)	
Pre-pregnancy BMI (kg/m^2^)	21.2 (19.4–23.7)	20.1 (18.6–39.1)	< 0.001
WBC (10^9^/L)	9.4 (8.0–10.9)	8.8 (7.6–10.1)	< 0.001
NEUT (%)	72.7 (68.5–76.7)	72.9 (68.7–76.4)	0.997
RBC (10^12^/L)	4.1 (3.8–4.3)	4.0 (3.7–4.3)	0.001
HGB (g/L)	122.0 (116.0–128.4)	121.8 (113.6–127.0)	0.005
HCT (%)	36.5 (34.5–38.3)	35.8 (33.6–37.8)	< 0.001
PLT (10^9^/L)	239.0 (205.6–276.1)	233.5 (199.9–268.0)	0.004
MPV (fL)	9.4 (8.5–10.2)	9.0 (8.1–9.9)	< 0.001
ALT (U/L)	13.0 (9.9–20.0)	12.0 (9.0–18.0)	0.003
AST (U/L)	16.0 (13.6–20.0)	16.0 (14.0–20.0)	0.621
Cr (μmol/L)	46.0 (41.4–51.0)	47.1 (42.0– 55.0)	< 0.001
Uric acid (μmol/L)	241.0 (207.7–241.0)	235.3 (201.5–269.0)	0.008
FBG (mmol/L)	4.6 (4.3–5.0)	4.5 (4.2–4.8)	< 0.001
PT (s)	12.2 (10.8–12.7)	12.4 (11.1–12.9)	< 0.001
APTT (s)	31.8 (28.6–34.3)	33.2 (29.6–35.2)	< 0.001
FIB (mg/dL)	4.2 (3.8–4.6)	4.1 (3.6–4.5)	< 0.001
PAPP-A (MoM)	1.0 (0.7–1.5)	1.1 (0.8–1.6)	< 0.001

*Abbreviations*: *GDM* Gestational diabetes mellitus, *DM* Diabetes mellitus, *PCOS* Polycystic ovary syndrome, *BMI* Body mass index, *WBC* White blood cell count, *NEUT* Neutrophil percentage, *RBC* Red blood cell count, *HGB* Hemoglobin, *HCT* Hematocrit, *PLT* Platelet count, *MPV* Mean platelet volume, *ALT* Alanine aminotransferase, *AST* Aspartate aminotransferase, *FBG* Fasting blood glucose, *PT* Prothrombin time, *APTT* Activated partial thromboplastin time, *FIB* Fibrinogen, *PAPP-A* Pregnancy associated plasma protein A

Variables that were significantly associated with GDM in univariate logistic regression were included in the multivariable logistic regression. The basic model and the extended model were constructed to identify potential risk factors associated with GDM (Table 5). The basic model showed that there were 10 independent predictors, including age, family history of diabetes, history of GDM, pre-pregnancy BMI, WBC, PLT, MPV, creatinine, uric acid, and FBG. The extended model showed that age, history of GDM, pre-pregnancy BMI, HCT, MPV, creatinine, uric acid, FBG, ALT, APTT, FIB, and PAPP-A were potential risk factors for GDM. The results of the collinearity test show that the variance inflation factors of all variables are less than 2, which preliminarily indicated that the problem of collinearity can be ignored.

**Table 5:** Multivariable logistic regression analysis based on the data from the training cohort

Characteristics	Basic model (n = 2895)			Extended model (n = 2116)		
	β-coefficient	*p*	OR (95%CI)	β-coefficient	*p*	OR (95%CI)
Age (year)	0.11	< 0.001	1.11 (1.08–1.14)	0.14	< 0.001	1.15 (1.11–1.18)
History of GDM, n (%)						
No	Reference			Reference		
Yes	1.92	< 0.001	6.80 (3.73–13.40)	1.84	< 0.001	6.27 (3.39–12.56)
Family history of DM, n (%)				Not included		
No	Reference					
Yes	2.36	< 0.001	10.60 (3.61–39.53)			
Pre-pregnancy BMI (kg/m^2^)	0.97	< 0.001	1.10 (1.07–1.14)	0.10	< 0.001	1.10 (1.06–1.15)
WBC (10^9^/L)	0.12	< 0.001	1.13 (1.08–1.20)	Not included		
PLT (10^9^/L)	0.00	0.021	1.00 (1.00–1.00)	Not included		
MPV (fL)	0.19	< 0.001	1.21 (1.12–1.31)	0.15	0.004	1.16 (1.05–1.29)
Creatinine (μmol/L)	-0.04	< 0.001	0.96 (0.95–0.98)	-0.02	< 0.001	0.98 (0.96–0.99)
Uric acid (μmol/L)	0.00	0.001	1.00 (1.00–1.01)	0.00	0.008	1.00 (1.00–1.00)
FBG (mmol/L)	0.39	< 0.001	1.47 (1.24–1.75)	0.48	< 0.001	1.61 (1.33–1.96)
HCT (%),	Not included			0.05	0.013	1.05 (1.01–1.10)
ALT (U/L)	Not included			0.01	0.014	1.01 (1.00–1.02)
APTT (s)	Not included			-0.07	< 0.001	0.93 (0.90–0.96)
FIB (mg/dL)	Not included			0.24	0.009	1.27 (1.06–1.52)
PAPP-A (MoM)	Not included			-0.27	0.002	0.76 (0.64–0.90)

*Abbreviations*: *CI* Confidence interval, *GDM* Gestational diabetes mellitus, *DM* Diabetes mellitus, *PCOS* Polycystic ovary syndrome, *BMI* Body mass index, *WBC* White blood cell count, *NEUT* Neutrophil percentage, *RBC* Red blood cell count, *HGB* Hemoglobin, *HCT* Hematocrit, *PLT* Platelet count, *MPV* Mean platelet volume, *ALT* Alanine aminotransferase, *AST* Aspartate aminotransferase, *FBG* Fasting blood glucose, *PT* Prothrombin time, *APTT* Activated partial thromboplastin time, *FIB* Fibrinogen, *PAPP-A* Pregnancy associated plasma protein A

### Nomograms and evaluation of nomograms

**Fig. 1:**
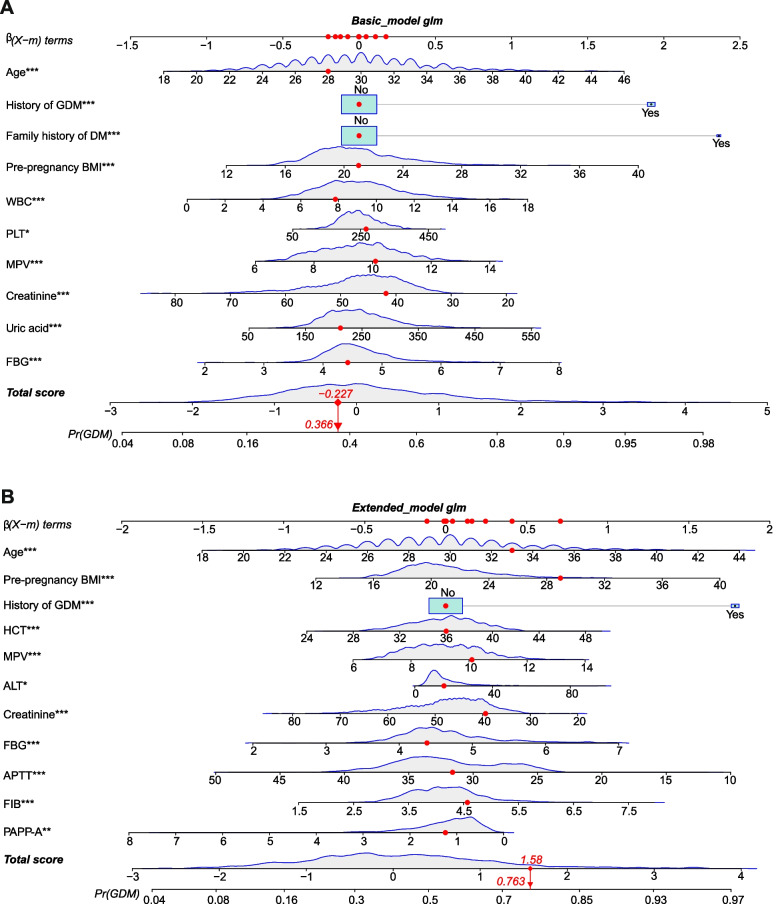
Nomogram for predicting probability of developing GDM of the basic model and extended model. ^*^p value between 0.01 and 0.05; ^**^p value between 0.0009 and 0.009; ^***^p value < 0.0009. Pre BMI, pre-pregnancy body mass index; GDM, gestational diabetes mellitus; FH, Family history of diabetes mellitus; FPG fasting plasma glucose; WBC, White blood cell count; PLT, Platelet count; MPV, Mean platelet volume; CR, Creatinine; UA, Uric acid; HCT, Hematocrit; ALT, Alanine aminotransferase; APTT, activated partial thromboplastin time; FIB, Fibrinogen; PAPP-A, pregnancy associated plasma protein A

Based on the outcomes of multivariable logistic analysis, a nomogram of the basic model was constructed using 10 factors (Fig. 1A), and a nomogram of the extended model was constructed using 11 factors (Fig. 1B). Take an example of nomogram usage (a participant was randomly selected from the extended population): 30 years old, no history of GDM, pre-pregnant BMI of 29 kg/m^2^, HCT of 36%, MPV of 10.1 fL, ALT of 15 U/L, creatinine of 39.9 μmol/L, FBG of 4.4 mmol/L, APTT of 31.5 s, FIB of 4.55 mg/dL, and PAPP-A of 1.250 MoM. Finally, a total score of 1.58 was obtained, and the corresponding incidence probability of GDM was 79.3%.

**Fig. 2:**
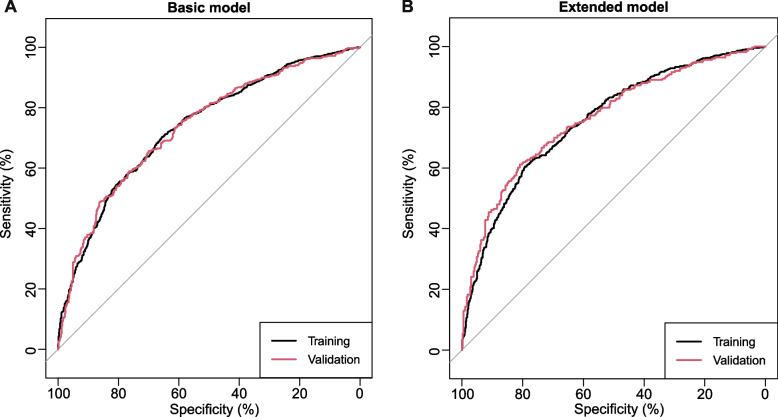
ROC curves for train and internal validation group in the basic model and in the extended model. **A** In the basic model, AUC of the train and internal validation group were 0.736 (95%CI: 0.71–0.76) and 0.736 (95%CI: 0.70–0.77). **B** In the basic model, AUC of the train and internal validation group were 0.756 (95%CI: 0.73–0.78) and 0.763 (95%CI: 0.73–0.80). ROC, receiver operating characteristic; AUC, area under the receiver operating characteristic

**Fig. 3:**
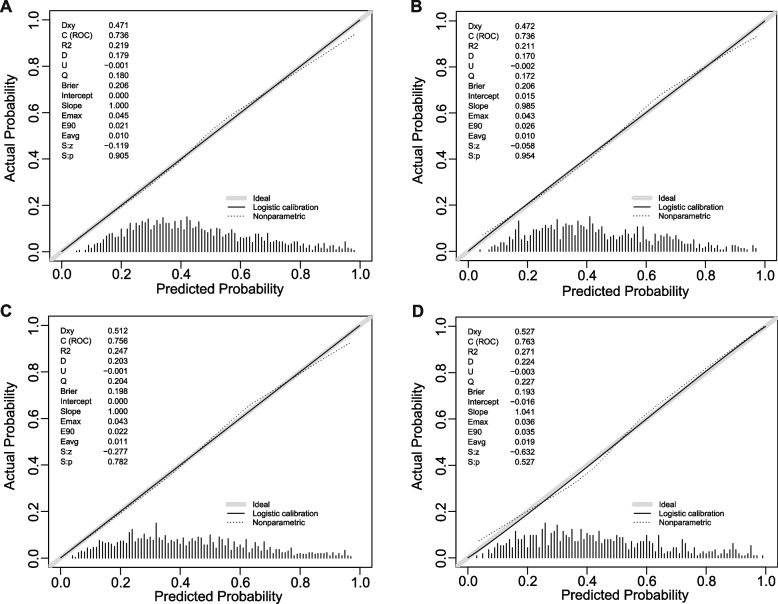
Calibration curves. **A** Basic model in the train cohort. **B** Basic model in the internal validation cohort. **C** Extended model in the train cohort. **D** Extended model in the internal validation cohort. Nomogram-predicted probability of GDM is plotted on the x-axis; actual probability of GDM is plotted on the y-axis. The line adjacent to the ideal line represents the predictive accuracy. GDM, gestational diabetes mellitus

**Fig. 4:**
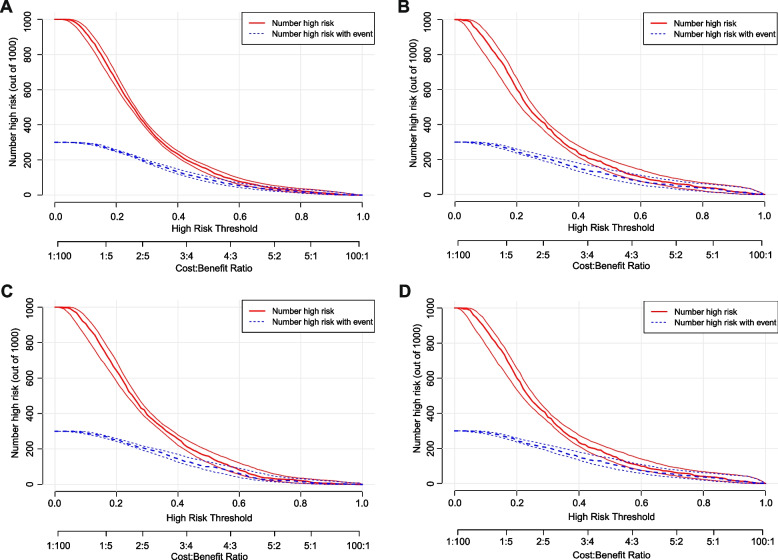
Clinical impact curves. **A** Basic model in the training cohort. **B** Basic model in the internal validation cohort. **C** Extended model in the training cohort. **D** Extended model in the internal validation cohort

In terms of discrimination, the nomogram of the basic model had an AUC of 0.736 (95%CI: 0.71–0.76) in the training cohort. Applying the exploratory set estimates to the internal validation set yielded an AUC of 0.736 (95%CI: 0.70–0.77) (Fig. 2A). The AUC of the extended model in the training cohort and the internal validation cohort was respectively 0.756 (95%CI: 0.73–0.78) and 0.763 (95%CI: 0.73–0.80) (Fig. 2B), indicating that the two models had good distinguishing abilities. The sensitivity and specificity of the internal validation of the basic model was 0.657 and 0.698, respectively, and those of the extended model was 0.612 and 0.809, respectively. The calibration of the nomogram was evaluated by Hosmer–Lemeshow test, and its calibrations curve had been drawn (Fig. 3A–D). The *p* value of the calibration curves of the basic model and the extended model in the training group was 0.289 and 0.636, respectively, and those in the internal validation was 0.684 and 0.635, respectively, indicating that the two models had good calibration abilities. To evaluate the clinical effects of the nomogram model more visually, the clinical impact curves were drawn. The “Number high risk” curve was closely to the “Number high risk with event” curve at high-risk threshold from 0.4 to 1.0, which indicated that the nomogram model owns extraordinary predictive power. The clinical impact curves of the basic model and the extended model showed that the predicted probability coincided well with the actual probability in the training cohort, respectively (Fig. 4A, B). Similar results were found in the validation cohort (Fig. 4C, D).

### Comparison and assessment of our model with the previous prediction models

**Fig. 5:**
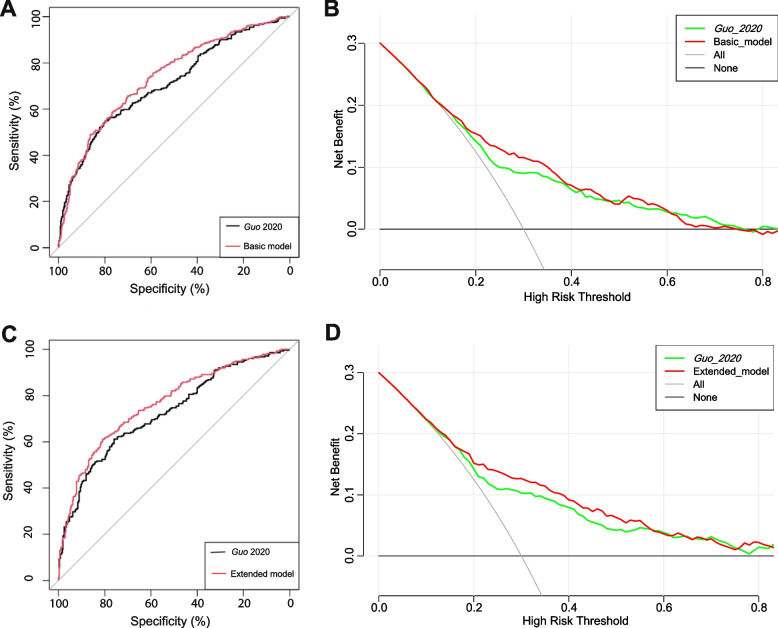
ROC curves and DCA. **A** Comparison between the basic model and the previous published model. **B** DCA of the basic model and the previous published model. **C** Comparison between the extended model and the previous published model. **D** DCA of the extended model and the previous published model. The horizontal line represented no cases will experience GDM; while the oblique line represents all cases will experience GDM. In the DCA, the area between the “black horizontal line” and “gray slope line” of the model curve meant the clinical validity of the model. The colorful lines represent the clinical net benefits according to the threshold probabilities. ROC, receiver operating characteristic curves; DCA, decision curve analysis; GDM, gestational diabetes mellitus

We compared the performance of our prediction models with a previously published early pregnancy prediction model based on the data in the present study, and this model was from *Guo 2020* [22] based on age, FBG, family history of diabetes, and history of GDM. The performances of the present nomograms comparing with the *Guo 2020* model are shown in Fig. 5A, C. According to the cross-validation, the performance of our basic model with an AUC of 0.736 (95%CI: 0.70–0.77) was greater than the *Guo 2020* prediction model with an AUC of 0.707 (95%CI: 0.67–0.74) (*p* = 0.024) (Fig. 5A). The performance of our extended model with an AUC of 0.763 (95%CI: 0.73–0.80) was also greater than the *Guo 2020* prediction model with an AUC of 0.726 (95%CI: 0.69–0.77) (*p* < 0.001) (Fig. 5C). The predictive abilities were confirmed by the significant AUC difference between our models and the previous published model (*p* < 0.05) (Table 6).

**Table 6:** Calibration and discrimination statistics of our basic model and the previous published model

Statistics	Basic model	*Guo 2020*	Extended model	*Guo 2020*
Calibration				
AIC	1052.298	1073.530	743.384	764.359
BIC	1104.739	1097.367	796.827	786.627
Discrimination				
NRI		*p* < 0.001		*p* < 0.001
IDI		*p* < 0.001		*p* < 0.001
AUC		*p* = 0.024		*p* < 0.001

*Abbreviations*: *AIC* Akaike information criterion, *BIC* Bayesian information criteria, *NRI* Net reclassification improvement, *IDI* integrated discrimination improvement, *AUC* Area under the receiver operating characteristic curve

The prediction performances of these models were also assessed by means of calibration and discrimination statistics. As showed in Table 6, there was little difference in AIC and BIC between our models and previous published model. In addition, the results for NRI and IDI are similar to those found for the ROC analysis: the risk factors of our basic model improve discrimination compared with the *Guo 2020* model (NRI = 0.364, 95%CI: 0.23–0.50, *p* < 0.001; IDI = 0.036, 95%CI: 0.02–0.05, *p* < 0.001). As well as the significance of NRI and IDI revealed that our extended model compared with the *Guo 2020* model (NRI = 0.386, 95%CI: 0.23–0.54, *p* < 0.001; IDI = 0.049, 95%CI: 0.03–0.07, *p* < 0.001), which can differentiate between pregnant women with subsequent GDM and normal pregnant women (Table 6).

The DCA plot indicated good positive net benefits of the predictive nomogram model among majority threshold probabilities (Fig. 5B, D). For example, at a threshold of 30% shown in Fig. 5D, the *Guo 2020* model would result in 11 per 100 participants being diagnosed with GDM, while our extended model will result in 13 per 100 participants being diagnosed with GDM without increasing false-positive results. In this analysis, all models showed a better cost effective than “treat all” and “treat none”, and our basic and extended models exhibited good performance.

## Discussion

We developed two novel models based on clinical characteristics and laboratory biomarkers, providing an estimation of patient-specific risks for GDM at the first antenatal care visit. The present study successfully developed and internally validated the basic and the extended model that could predict the risk of developing GDM among patients with a singleton pregnancy before 16 weeks of gestation. The six predictors determined were age, history of GDM, pre-pregnancy BMI, FBG, creatinine, and MPV, which were all tested in our two models. Notably, the extended model that introduces APTT, FIB, and PAPP-A had a greater AUC of 0.763 compared with the basic model with an AUC of 0.736 for the same internal validation cohort. The nomogram demonstrated a favorable calibration for predicting the probability of GDM. When comparing with *Guo 2020* model, our nomograms performed greater in calibration and discrimination*.* In addition, we performed a DCA to quantify the clinical usefulness of the models and found that our basic and extended models are greater.

We integrated other classical indicators associated with GDM to develop a prediction model in the early pregnancy. Consistent with previous findings [23–25], the present study showed that age, the proportion of history of GDM, the proportion of family history of DM, pre-pregnancy BMI, and FBG level of the women with GDM were significantly higher than those of normal pregnant women. A previous study found that the levels of WBC, PLT, and ALT in the early pregnancy were positively correlated with the risk of developing GDM, but the increased level of creatinine corresponded to a reduced risk [26]. The present results are concordant with several previous findings reporting that the level of MPV of patients with GDM was higher than that of the normal pregnant women [27]. Zhao et al. [28] found that serum uric acid was positively related to insulin resistance and increased in the patients with GDM in the early pregnancy, which is consistent with the present study. The same as the present result, HCT was reported to be significantly higher in the women with GDM in the early pregnancy [29]. Additionally, the present study demonstrated that the level of APTT of women with GDM in the early pregnancy was lower, whereas the level of FIB was higher in GDM group, suggesting that patients with GDM may be hypercoagulable. Those changes of laboratory biomarkers indicated that the pregnant women who subsequently develop GDM may have some abnormal functional changes during the early pregnancy.

Additionally, we found a lower level of PAPP-A in the women with GDM in the early pregnancy, which is consistent with the findings from Tenenbaum-Gavish *et al*. [18]. PAPP-A MoM refers to circulating PAPP-A concentrations adjusted by diverse maternal factors and medical history, such as age, weight, smoking, race, and diabetes status [30]. The decreased PAPP-A MoM of patients with GDM in the early pregnancy may be associated with impaired adipose tissue remodeling, enhanced pregnancy-induced insulin resistance, and impaired glucose tolerance [31]. Therefore, PAPP-A level in the early pregnancy may be important for GDM prediction. To the best of our knowledge, the introduction of the PAPP-A MoM into the nomogram has not been previously reported.

Although several biomarkers, such as adiponectin, plasma fatty acid-binding protein 4, and sex hormone binding globulin, can also achieve a great AUC [11, 12, 32], and various first-trimester prediction models for GDM have been proposed [33–35], no specific method has been widely used in routine clinical practice. The possible reasons may be that some biomarkers are not routinely tested for every pregnant woman, and some advanced technologies, such as machine learning, are not widely used in routine clinical practice. Therefore, it is necessary to explore more accurate and applicable approaches for predicting GDM in the early pregnancy.

In the present study, the two nomograms are adapted to difference hospital situations, which integrating multiple routine factors during the early pregnancy. The aim of the present study is to detect as many pregnant women with subsequent GDM as possible with high coverage of laboratory parameters and without increasing the psychological and economic burden. Meanwhile, the nomograms showed favorable discrimination and clinical usability for predicting the probability of GDM. However, we need to perform an external validation for the models using a multicenter prospective study in the further.

## Conclusions

Early warning and early intervention are of great significance to the prevention, intervention, and prognosis of GDM. The present study established two applicable nomogram models that may accurately predict the risk of developing GDM when the first antenatal visit (generally in the early pregnancy).

## Supplementary Information

**Additional file 1.**

**Additional file 2.**

## Data Availability

All data generated or analyzed during this study are included in the supplementary information files.

## Abbreviations

ALT: Alanine aminotransferase
APTT: Activated partial thromboplastin time
AST: Aspartate aminotransferase
AUC: Area under the receiver operating characteristic curve
BMI: Body mass index
DCA: Decision-curve analysis
DM: Diabetes mellitus
FBG: Fasting blood glucose
FIB: Fibrinogen
GDM: Gestational diabetes mellitus
HCT: Hematocrit
HGB: Hemoglobin
IDI: Integrated discrimination improvement
MPV: Mean platelet volume
NEUT: Neutrophil percentage
NRI: Net reclassification improvement
OGTT: Oral glucose tolerance test
PAPP-A: Pregnancy associated plasma protein A
PCOS: Polycystic ovary syndrome
PLT: Platelet count
PT: Prothrombin time
RBC: Red blood cell count
ROC: Receiver operating characteristic
WBC: White blood cell count
